# Nonsymmorphic symmetry protected node-line semimetal in the trigonal YH_3_

**DOI:** 10.1038/s41598-018-19870-5

**Published:** 2018-01-23

**Authors:** Dexi Shao, Tong Chen, Qinyan Gu, Zhaopeng Guo, Pengchao Lu, Jian Sun, Li Sheng, Dingyu Xing

**Affiliations:** National Laboratory of Solid State Microstructures, School of Physics and Collaborative Innovation Center of Advanced Microstructures, Nanjing University, Nanjing, 210093 China

## Abstract

Using ab initio calculations based on density-functional theory and effective model analysis, we propose that the trigonal YH_3_ (Space Group: P$\bar{{\bf{3}}}$c1) at ambient pressure is a node-line semimetal when spin-orbit coupling (SOC) is ignored. This trigonal YH_3_ has very clean electronic structure near Fermi level and its nodal lines locate very closely to the Fermi energy, which makes it a perfect system for model analysis. Symmetry analysis shows that the nodal ring in this compound is protected by the glide-plane symmetry, where the band inversion of |*Y*^+^, *d*_*xz*_〉 and |*H*1^−^, *s*〉 orbits at Γ point is responsible for the formation of the nodal lines. When SOC is included, the line nodes are prohibited by the glide-plane symmetry, and a small gap (≈5 meV) appears, which leads YH_3_ to be a strong topological insulator with Z_2_ indices (1,000). Thus the glide-plane symmetry plays an opposite role in the formation of the nodal lines in cases without and with SOC. As the SOC-induced gap is so small that can be neglected, this P$\bar{{\bf{3}}}$c1 YH_3_ may be a good candidate for experimental explorations on the fundamental physics of topological node-line semimetals. We find the surface states of this P$\bar{{\bf{3}}}$c1 phase are somehow unique and may be helpful to identify the real ground state of YH_3_ in the experiment.

## Introduction

Topological semimetals (TSMs) have attracted great attention for both theoretical interests and experimental applications in recent years. Different from time-reversal symmetry (TRS) protected Z_2_ topological insulators (TIs)^1,2^ which are insulating in the bulk, TSMs are materials where the conduction and the valence bands cross with each other at certain locations in the Brillouin Zone (BZ). Usually, the band crossing is protected by certain symmetries, i.e., perturbations on the Hamiltonian which respect the symmetries can not break the crossing. Recently, several types of TSMs have been proposed to investigate the fermion-like excitations, including Dirac fermions^3–6^, Weyl fermions^7–10^ and nodal lines^11–16^. These compounds are named as: Dirac semimetals (DSMs), Weyl semimetals(WSMs) and node-line semimetals (NLSMs), respectively.

Up to now, there have been a lot of reports about the progress in DSMs and WSMs, for example, 3D Dirac semimetals have recently been identified experimentally in Cd_3_As_2_^17–21^ and Na_3_Bi systems^22,23^. Similarly, TaAs^9,24–26^, NbAs^27^, TaP^28^, WTe_2_^29,30^, and MoTe_2_^31^ etc are verified to be WSMs experimentally in recent years. Different from DSMs and WSMs, in which the conduction and valence bands touch at discrete points, the crossings of NLSMs form a closed loop in the BZ. Although many candidates of NLSMs have been proposed and much efforts has been made to investigate them, the corresponding progress in the experiment is slow, because an open surface usually breaks the inversion or some mirror symmetries which are important to the formation of nodal lines^32^.

Materials experimentally confirmed (or partially confirmed) to host nodal line include Be metal^16^, ZrSiS^33^, PbTaSe_2_^14^ and ZrSiSe/ZrSiTe^34^. Therefore, theoretical predictions on more candidates of node-line semimetal are still in demand. It is well known that symmetries play important roles in identifying various of TIs and topological superconductors (TSCs)^35–39^. In fact, symmetries are also important in classifying NLSMs, for instance, three types of NLSMs protected by different symmetries have been proposed: (a) mirror symmetry protected NLSMs^8,14,16,40^, (b) coexistence of TRS and inversion symmetry (IS) protected NLSMs^11–13,32^ and (c) nonsymmorphic symmetry protected NLSMs^15,32^.

Hydrides is a large class of materials and has been extensively investigated in many aspects, including energy storage^41^ and superconductivity^42–46^, etc. Since Ashcroft proposed that high *T*_*c*_ superconductivity can be obtained in hydrogen and hydrides under high pressure^47,48^, many hydrides have been investigated. Yttrium-hydrogen system becomes interesting due to the same reason. For instance, a fcc YH_3_ has been predicted to be a superconductor with *T*_*c*_ ~ 40 K at 17.7 GPa^49^. Later work predicts that two new yttrium hydrides, i.e., YH_4_ and YH_6_, are also superconductors with *T*_*c*_ ~ 84–95 K and *T*_*c*_ ~ 251–264 K at 120 GPa, respectively^50^. Very recently, YH_10_ in the space group of both $Im\bar{3}m$ and $Fm\bar{3}m$ has been predicted to be a room-temperature superconductor under very high pressure^51,52^.

Though many works about superconductivity of yttrium-hydrogen system under pressure have been implicated, very few explorations on the topological electronic properties of hydrides have been reported so far^53–55^. In this work, we predicted that YH_3_ in the space group of P$\bar{3}$c1 at ambient pressure is a node-line semimetal when spin-orbit coupling (SOC) is ignored. Especially, the YH_3_ system we studied has extremely clean electronic structures near the Fermi level; i.e., there are no other pockets. The energy of the crossing points along the nodal loop varies within a very small energy range, from around −5 to 35 meV. Therefore, this nodal loop is very “flat” in the energy and momentum space, which makes YH_3_ a perfect model system for NLSMs. In general, NLSMs without SOC will transform into either insulators, DSMs, WSMs or even double NLSMs when SOC is considered, which is much related to the symmetries in the corresponding systems^56^. While in this work, when SOC is included, the three nodal lines around Γ point will be gapped out with a small gap (≈5 meV), making YH_3_ a topological insulator with Z_2_ = (1,000). Nevertheless, further calculation shows that the gap induced by SOC along the nodal ring is very small (about 5 meV), which indicates that the effect of SOC is negligible and the characteristic of the nodal ring can be preserved.

## Methods

Calculations of the band structures are performed using the full-potential linearized augmented plane-wave (FP-LAPW) method^57,58^ implemented in the WIEN2k^59^ package. We use 13 × 13 × 11 k-mesh for the BZ sampling and −7 for the plane wave cut-off parameter R_*MT*_K_*max*_ for the electronic structure calculation, where the R_*MT*_ is the minimum muffin-tin radius and K_*max*_ is the plane-wave vector cut-off parameter. SOC is taken into consideration by a second-variation method^60^. The tight-binding models are constructed with the maximally localized Wannier functions (MLWFs) method^61–63^, the corresponding hopping parameters are determined from the projections of the bulk Bloch wave functions. The projected surface states are calculated using surface Green’s function in the semi-infinite system^64,65^.

## Results and Discussions

### The crystal structure of YH_3_

Historically, three different phases of YH_3_ have been reported to be the ground state potentially. Two of them are experimentally favoured with trigonal P$\bar{3}$c1 and hexagonal P6_3_cm symmetry^66–71^, while the third candidate is in the space group of P6_3_ which was predicted theoretically^72^. It seems that the ground state of YH_3_ at ambient pressure is still under debate because the three candidates have very tiny total energy difference (0.001 eV/f.u.) from theoretical point of view. First, the hexagonal P6_3_ structure is only theoretically proposed and seems to disagree with the neutron-diffraction results^73,74^. Second, later neutron-diffraction experiments^71,75^ identify that the P$\bar{3}$c1 structure is stable from the ambient pressure up to 12 GPa. Thus, in the following, we only focus on the phase of YH_3_ with the P$\bar{3}$c1 symmetry.

**Figure 1:**
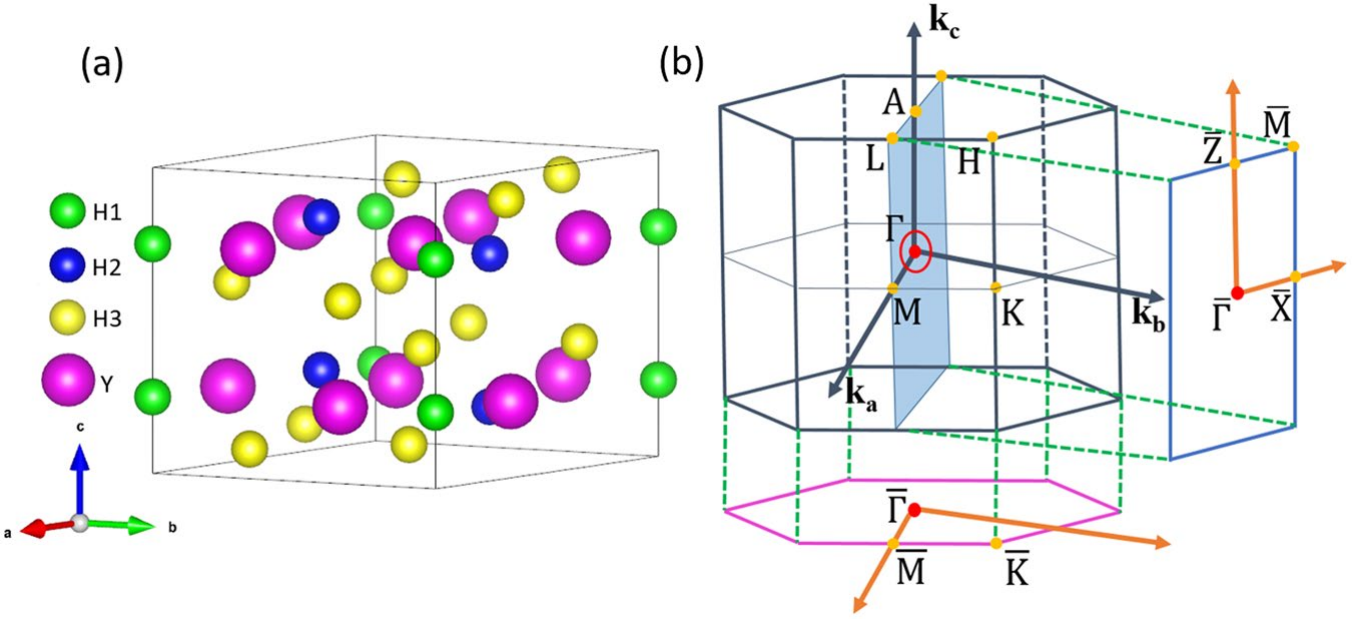
(**a**) Crystal structure of YH_3_ at the ambient pressure with P$\bar{3}$c1 symmetry. H1, H2 and H3 atoms occupy the 2a $(0,0,\tfrac{1}{4})$, 4d $(\tfrac{1}{3},\tfrac{2}{3},0.181)$ and 12 g (0.348, 0.025, 0.093) sites, respectively, while Y atoms lie at the 6f $(0.336,0,\tfrac{1}{4})$ sites. (**b**) The corresponding BZ and its projection onto the (010) direction. The red ring on the shadow plane surrounding the Γ point represents the node-line structure in the BZ.

The crystal structure and corresponding BZ of P$\bar{3}$c1 (Space Group No. 165) YH_3_ is shown in Fig. 1(a,b) respectively. We use the experimental lattice parameters from literature^71^ in our calculations, which are listed in Table 1.

**Table 1:** The experimental lattice parameters of YH_3_ with P$\overline{3}$c1 symmetry^71^.

Phase	a = b	c	*α* = *β*	*γ*
P$\overline{3}$c1	6.359 (*Å*)	6.607 (*Å*)	90°	120°

### Band structures without SOC and the corresponding model analysis

**Figure 2:**
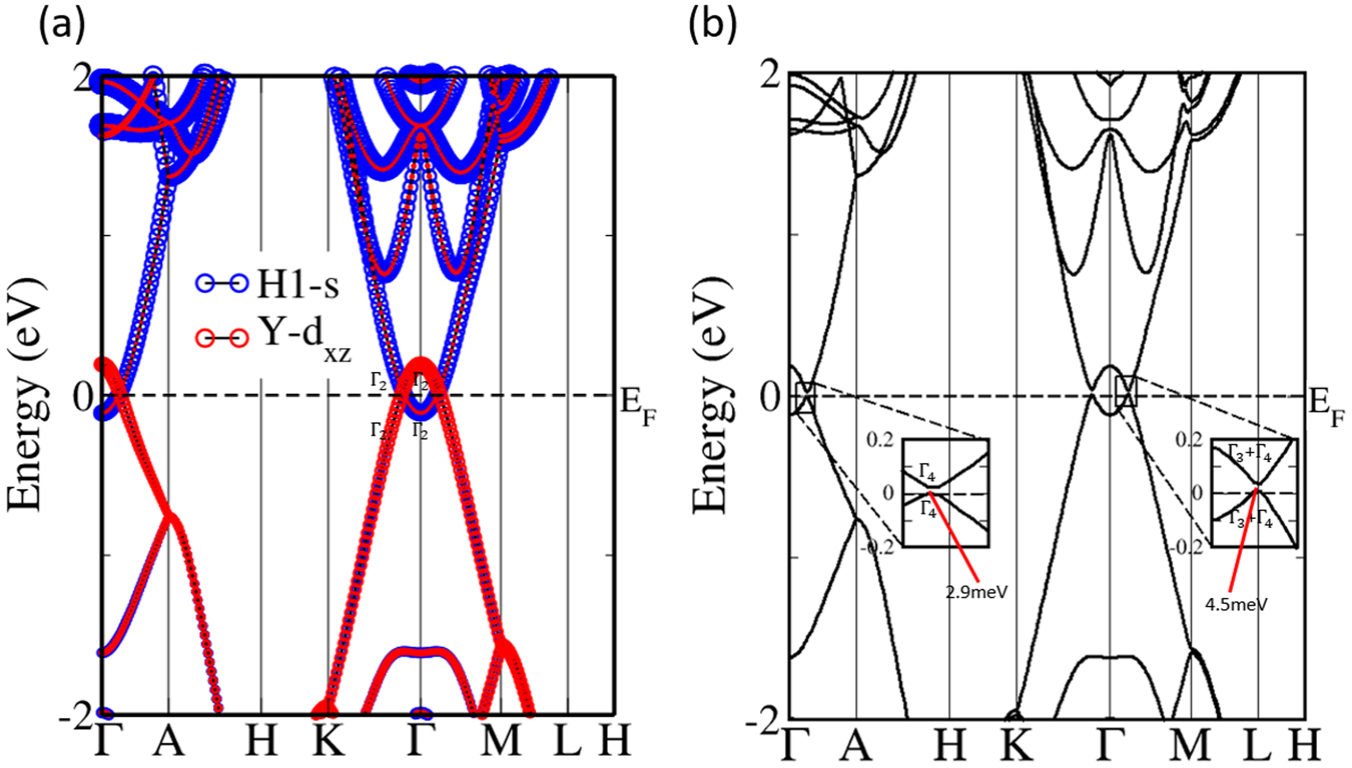
(**a**) Corresponding fat-band structure of YH_3_ in the space group of P$\bar{3}$c1 without SOC. The bands between CBM and VBM is gapped along *K* → Γ as the irreducible representations have showed. (**b**) Corresponding band structure of YH_3_ in the space group of P$\bar{3}$c1 with SOC.

From the band structures of P$\bar{3}$c1 YH_3_ without SOC shown in Fig. 2(a), we can find three Dirac crossings composed of the conduction band minimum (CBM) and the valence band maximum (VBM) near Γ along the high-symmetry path in the BZ at the first sight. Detailed first-principle calculations indicates that Dirac crossings lying in the plane *m*_Γ–*M*–*L*–*A*_ is protected by the *G*_*x*_ symmetry, while Dirac crossing along *K* → Γ is not symmetry-protected (both the irreducible representations of CBM and VBM are Γ_2_) i.e., hybridization between the CBM and the VBM will open a gap in-between. Further calculations indicates that the CBM and the VBM are contributed mainly by Y-d_*xz*_ and H1-s orbits, respectively, as the fat-band showed in Fig. 2(a). The band inversion (not caused by SOC) of Y-d_*xz*_ and H1-s at Γ point leads to a gap of 0.302 eV.

To give more insights of the nodal lines surrounding the Γ point, we have established an effective Hamiltonian by **k** · **p** method. Taking the crystal symmetry and TRS into consideration, the effective Hamiltonian can be written as follows:

1 $$\begin{array}{rcl}H(\overrightarrow{k}) & = & {g}_{0}(\overrightarrow{k}){\tau }_{0}+{g}_{x}(\overrightarrow{k}){\tau }_{x}+{g}_{z}(\overrightarrow{k}){\tau }_{z}\\ {g}_{0}(\overrightarrow{k}) & = & {M}_{0}-{B}_{0}({k}_{x}^{2}+{k}_{y}^{2})-{C}_{0}{k}_{z}^{2}\\ {g}_{x}(\overrightarrow{k}) & = & A({k}_{x}^{3}-3{k}_{x}{k}_{y}^{2})\\ {g}_{z}(\overrightarrow{k}) & = & {M}_{z}-{B}_{z}({k}_{x}^{2}+{k}_{y}^{2})-{C}_{z}{k}_{z}^{2}.\end{array}$$

Here, the *τ*_*x*_ and *τ*_*z*_ are Pauli matrices, *τ*_0_ is a 2 × 2 unit matrix. This system has both TRS and IS, thus, the component of *τ*_*y*_ must be zero^11^. We can obtain the eigenvalues of the two-level system by diagonalizing the 2 × 2 effective Hamiltonian and the results are $E(\overrightarrow{k})={g}_{0}(\overrightarrow{k})\pm \sqrt{{g}_{x}^{2}+{g}_{z}^{2}}$. Band crossings of the nodal lines will occur when *g*_*x*_ = *g*_*z*_ = 0. It’s clear that ${g}_{z}(\overrightarrow{k})=0$ gives us *M*_*z*_*B*_*z*_ > 0 and *M*_*z*_*C*_*z*_ > 0. We find that *M*_*z*_*B*_*z*_ > 0 and *M*_*z*_*C*_*z*_ > 0 are exactly the condition of band inversion. Furthermore, ${g}_{x}(\overrightarrow{k})=0$ confines the band crossings of the node-line in the *k*_*x*_ = 0, ±$\sqrt{3}{k}_{y}$ planes, i.e., there are three nodal rings surrounding the Γ point and lying in mirror-invariant planes *m*_Γ–*M*–*L*–*A*_, as shown with the shadow sector in Fig. 1(b). It’s obvious that these three nodal rings are related to each other by *R*_3*z*_ symmetry.

### Band structures with SOC and the corresponding model analysis

When SOC is considered, band crossings of the three nodal lines will disappear, as the corresponding band structure with SOC shown in Fig. 2(b). We will further explain the above-mentioned phenomenon in the following. Taking SOC into account, spin and orbital angular momentum are coupled together, which generates a group of new eigenstates with certain total angular momentum quantum numbers. Then we can mark these new eigenstates of the CBM and VBM as $|H{1}_{s}^{-},\pm \frac{1}{2}\rangle$, and $|{Y}_{{d}_{xz}}^{+},\pm \frac{1}{2}\rangle$. Here subscripts s and d_*xz*_ denote corresponding orbits consisting of the new eigenstates and the superscripts ± represent the parities of corresponding eigenstates, respectively.

According to the analysis of irreducible representations and projected orbits, the CBM and VBM at the Γ point (denoted as ${{\rm{\Gamma }}}_{4}^{-}$ and ${{\rm{\Gamma }}}_{4}^{+}$) are mainly composed of $|H{1}_{s}^{-},\pm \frac{1}{2}\rangle$ and $|{Y}_{{d}_{xz}}^{+},\pm \frac{1}{2}\rangle$ basis, respectively. If we arrange the 4 basis in the order of $|H{1}_{s}^{-},\frac{1}{2}\rangle$, $|H{1}_{s}^{-},-\frac{1}{2}\rangle$, $|{Y}_{{d}_{xz}}^{+},\frac{1}{2}\rangle$, $|{Y}_{{d}_{xz}}^{+},-\frac{1}{2}\rangle$, and then take the time-reversal and *D*_3*d*_ point-group symmetries at the Γ point into consideration, we can give the character table of Γ matrices and the polynomials of momentum $\overrightarrow{k}$ for this system as shown in Table 2.

**Table 2:** The character table for the P$\overline{3}$c1 YH_3_.

Γ	Representation	T/P	$\overrightarrow{{\boldsymbol{k}}}$
Γ_1_	${\tilde{{\rm{\Gamma }}}}_{1}^{+}/{A}_{1g}$	+	$1,{k}_{x}^{2}+{k}_{y}^{2},{k}_{z}^{2}$
Γ_2_	${\tilde{{\rm{\Gamma }}}}_{2}^{-}/{A}_{2u}$	−	${k}_{z},{k}_{z}^{3},3{k}_{x}^{2}{k}_{y}-{k}_{y}^{3}$
Γ_3_	${\tilde{{\rm{\Gamma }}}}_{1}^{-}/{A}_{1u}$	−	${k}_{x}^{3}-3{k}_{x}{k}_{y}^{2}$
{Γ_4_, Γ_5_}	${\tilde{{\rm{\Gamma }}}}_{3}^{-}/{E}_{u}$	−	$\{{k}_{x},{k}_{y}\},\{{k}_{x}^{3}+{k}_{x}{k}_{y}^{2},{k}_{x}^{2}{k}_{y}+{k}_{y}^{3}\}$

From Table 2, our model Hamiltonian yields as

2 $$\begin{array}{rcl}H(\overrightarrow{k}) & = & {\varepsilon }_{0}(\overrightarrow{k})+\sum _{i=1}^{5}\,{f}_{i}(\overrightarrow{k}){{\rm{\Gamma }}}_{i}\\  & = & {\varepsilon }_{0}(\overrightarrow{k})+(\begin{array}{cccc}M(\overrightarrow{k}) & 0 & A(\overrightarrow{k}) & C(\overrightarrow{k})\\ 0 & M(\overrightarrow{k}) & {C}^{\ast }(\overrightarrow{k}) & B(\overrightarrow{k})\\ {A}^{\ast }(\overrightarrow{k}) & C(\overrightarrow{k}) & -M(\overrightarrow{k}) & 0\\ {C}^{\ast }(\overrightarrow{k}) & {B}^{\ast }(\overrightarrow{k}) & 0 & -M(\overrightarrow{k})\end{array})\end{array}$$

which describes the dispersion of the CBM and VBM around the Γ point. Here we use the following Γ matrices:

3 $$\begin{array}{lll}{{\rm{\Gamma }}}_{1}={\sigma }_{3}\otimes {\tau }_{0} & {{\rm{\Gamma }}}_{2}={\sigma }_{1}\otimes {\tau }_{3} & {{\rm{\Gamma }}}_{3}={\sigma }_{2}\otimes {\tau }_{0}\\ {{\rm{\Gamma }}}_{4}={\sigma }_{1}\otimes {\tau }_{1} & {{\rm{\Gamma }}}_{5}={\sigma }_{1}\otimes {\tau }_{2}, & \end{array}$$

which satisfy the Clifford algebra {Γ_*a*_, Γ_*b*_} = 2*δ*_*ab*_. While the other ten Γ matrices are given by ${{\rm{\Gamma }}}_{ab}=\frac{1}{2i}[{{\rm{\Gamma }}}_{a},{{\rm{\Gamma }}}_{b}]$. Presence of both TRS and IS will forbid the existence of these ten Γ_*ab*_ terms in our model Hamiltonian. In Equation (2), ${\varepsilon }_{0}(\overrightarrow{k})={D}_{0}-{m}_{0}{k}_{z}^{2}$ − ${n}_{0}({k}_{x}^{2}+{k}_{y}^{2})$, $M(\overrightarrow{k})={D}_{1}-{m}_{1}{k}_{z}^{2}$ − ${n}_{1}({k}_{x}^{2}+{k}_{y}^{2})$, $A(\overrightarrow{k})={D}_{2}{k}_{z}+{E}_{2}{k}_{z}^{3}$ + ${F}_{2}\mathrm{(3}{k}_{x}^{2}{k}_{y}-{k}_{y}^{3})$ − $i{D}_{3}({k}_{x}^{3}-3{k}_{x}{k}_{y}^{2})$, $B(\overrightarrow{k})=-{D}_{2}{k}_{z}-{E}_{2}{k}_{z}^{3}$ − ${F}_{2}\mathrm{(3}{k}_{x}^{2}{k}_{y}-{k}_{y}^{3})$ − $i{D}_{3}({k}_{x}^{3}-3{k}_{x}{k}_{y}^{2})$ and $C(\overrightarrow{k})={D}_{45}{k}_{-}$ + ${E}_{45}({k}_{x}^{2}+{k}_{y}^{2}){k}_{-}$ with *k*_−_ = *k*_*x*_ − *ik*_*y*_.

From the model Hamiltonian given in Equation (2) together with the band structure shown in Fig. 1(b), we can draw some conclusions as the following. First of all, ${\varepsilon }_{0}(\overrightarrow{k})$ will break the particle-hole symmetry for the CBM and VBM around the Γ point. Secondly, *D*_1_ in $M(\overrightarrow{k})$ will lead to a gap at the Γ point. Thirdly, to reproduce band inversion, we need that ${D}_{1}{m}_{1} > 0\cap {D}_{1}{n}_{1} > 0$. More importantly, the dispersions of the model Hamiltonian given by Equation (2) are $E(\overrightarrow{k})={f}_{0}(\overrightarrow{k})\pm \sqrt{{f}_{1}^{2}(\overrightarrow{k})+{f}_{2}^{2}(\overrightarrow{k})+{f}_{3}^{2}(\overrightarrow{k})+{f}_{4}^{2}(\overrightarrow{k})+{f}_{5}^{2}(\overrightarrow{k})}$ and both dispersions are doubly degenerate. As a result, a band crossing of this model requires *f*_1_ = *f*_2_ = *f*_3_ = *f*_4_ = *f*_5_ = 0. Several discrete $\overrightarrow{k}$ points near the Γ point may satisfy the above-mentioned conditions. For example, $\overrightarrow{k}=(0,0,\pm \sqrt{-\frac{{D}_{2}}{{E}_{2}}})$ when ${D}_{2}{E}_{2} < 0\cap \frac{{D}_{1}}{{m}_{1}}=-\frac{{D}_{2}}{{E}_{2}}$ stands. It means that we may find some Dirac crossings at the first sight. However, on one hand, $\frac{{D}_{1}}{{m}_{1}}=-\frac{{D}_{2}}{{E}_{2}}$ is a very rigorous condition and can not be obtained without other symmetries. More importantly, on the other hand, we can explain that $\overrightarrow{k}$ points lying in the plane *m*_Γ–*M*–*L*–*A*_ must induce a gap in the following. There are three generator operators existing in the nonsymmorphic space group P$\overline{3}$c1, we sign the three-fold rotation axis around z axis, the glide plane located at x = 0, and the inversion symmetry as R_3*z*_, G_*x*_ and P, respectively. The operation of G_*x*_ acts in both the real space (*x*, *y*, *z*) and the spin space as

4 $$\begin{array}{rcl}{G}_{x}\,:(x,y,z) & \to  & (-x,y,z+\tfrac{1}{2})\\ {G}_{x}\,:({s}_{x},{s}_{y},{s}_{z}) & \to  & ({s}_{x},-{s}_{y},-{s}_{z}\mathrm{)}.\end{array}$$

Similar with the analysis in the work by Fang *et al*.^32^, we can easily find

5 $${G}_{x}\ast (P\ast T)={e}^{-i{k}_{z}}(P\ast T)\ast {G}_{x}.$$

On the mirror invariant plane *k*_*x*_ = 0, the bands can be labeled by its *G*_*x*_ eigenvalues. When SOC is considered, we have

6 $${G}_{x}^{2}=-{e}^{-i{k}_{z}}$$

the minus sigh is because ${G}_{x}^{2}$ includes a 2Π rotation in the spin space, which gives a −1 for a spin − $\tfrac{1}{2}$ system. So the eigenvalue of *G*_*x*_ is ± $i{e}^{-i\frac{{k}_{z}}{2}}$. The existence of both P and T will ensure all bands locally degenerated at every $\overrightarrow{k}$ point in the BZ when SOC is considered, and the degenerated bands are related to each other by P $\ast$ T. Suppose at (0, *k*_*y*_, *k*_*z*_), a Bloch function $|\psi (\overrightarrow{k})\rangle$ is an eigenstate of *G*_*x*_ with eigenvalue $i{e}^{-i\frac{{k}_{z}}{2}}$, then its degenerate partner $P\ast T|\psi (\overrightarrow{k})\rangle$ under *G*_*x*_,

7 $$\begin{array}{rcl}{G}_{x}(P\ast T)|\psi (\overrightarrow{k})\rangle  & = & {e}^{-i{k}_{z}}(P\ast T){G}_{x}|\psi (\overrightarrow{k})\rangle \\  & = & {e}^{-i{k}_{z}}(P\ast T)i{e}^{-i\frac{{k}_{z}}{2}}|\psi (\overrightarrow{k})\rangle \\  & = & -i{e}^{-i\frac{{k}_{z}}{2}}(P\ast T)|\psi (\overrightarrow{k})\rangle .\end{array}$$

It means that the degenerated bands on the *k*_*x*_ = 0 plane have opposite *G*_*x*_ eigenvalues, and two sets of such doublet bands generally anticross, i.e., the bands with the same *G*_*x*_ eigenvalues hybridize and avoid crossing. In other words, nodal lines near the Γ point in the *k*_*x*_ = 0 plane (without SOC) will disappear in the whole BZ when SOC is considered, and *G*_*x*_ symmetry plays the key role of prohibiting the band crossing between CBM and VBM, even though the gap is very small (≈5 meV) as the **k** · **p** Hamiltonian in Equation (2) has showed. As a result, this node-line semimetal will become an insulator when SOC is considered. With both TRS and IS in this system, we can easily calculate the *Z*_2_ index by multiplying all the parities of all the occupied bands at all time-reversal-invariant momenta (TRIMs) using the method by Fu and Kane^76^. The results are shown in Table 3, which indicates the P$\bar{3}$c1 YH_3_ is a strong TI with *Z*_2_ = (1,000) when SOC is taken into consideration. Nevertheless, the effect of the SOC is negligible because the H atom is small and the Y atom is also not very heavy; there is only one d electron in the Y atom. We have performed calculations with SOC and found that the SOC induced gap along the nodal ring is very small (about 5 meV), which indicates that the effect of SOC could be ignored and the characteristic of the nodal ring can be preserved.

**Table 3:** The parities product of all the occupied bands at the eight TRIMs for the P$\bar{3}$c1 phase of YH_3_.

TRIM	Γ	3 M	A	3 L;	Total
Parity	+	−	−	−;	−

### Surface states with and without SOC

**Figure 3:**
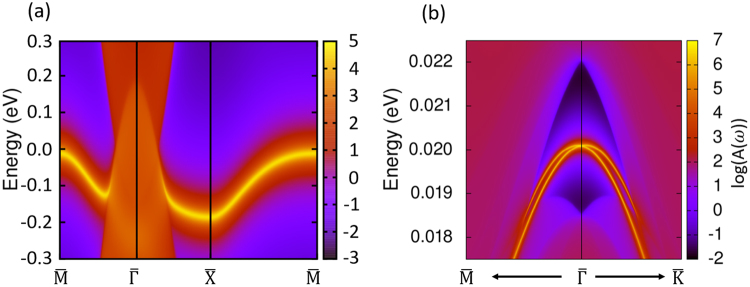
(**a**) The surface states without SOC of P$\overline{3}$c1 YH_3_ terminated with H atoms in the 〈010〉 direction, (**b**) The surface states with SOC of P$\overline{3}$c1 YH_3_ terminated with H atoms in the 〈001〉 direction.

Exotic topological surface states are an important property to identify various topological phases. Based on the tight-binding model constructed with WLWFs and surface Green function methods^64,65,77^, we have calculated the 〈010〉 surface states of the P$\bar{3}$c1 YH_3_ without SOC and the 〈001〉 surface states with SOC, as shown in Fig. 3(a,b), respectively. In particular, as the nice picture shows in Fig. 3(a), topological protected surface states without SOC signed with a bright curve connects the nodal points across the boundary of BZ. When SOC is included, the gap along Γ → *A* is so small (≈5 meV) that we may consider the CBM and VBM are nearly touched in the bulk band structure, this phenomenon can be proved that we can find the corresponding 〈001〉 surface states connecting the psudo-touch points from Fig. 3(b). We think these topological protected surface states may be helpful to identify the real ground state of YH_3_ from those three candidates. For example, we propose that angle-resolved photoemission spectroscopy (ARPES) technique can be used to investigate these surface states of this node-line semimetal candidate. If a bright surface state could be found in the 〈010〉 direction, and two parabolic bright curves with negative mass touching at the $\bar{{\rm{\Gamma }}}$ point could be found in the 〈001〉 direction, then the YH_3_ sample should be in the P$\bar{3}$c1 symmetry.

## Conculsion

In conclusion, based on first-principles calculations and effective model analysis, we propose that the P$\overline{3}$c1 YH_3_ is a nonsymmorphic symmetry protected node-line semimetal when SOC is ignored. This system has very clean electronic structures, there are no other pockets except the ones composing the node line near the Fermi level. The energy of the crossing points along the nodal loop has a very small energy range, from around −5 to 35 meV. Therefore, this nodal loop is very “flat” in the energy and momentum space, which makes YH_3_ a perfect system for model analysis. There are three node-lines related to each other by *R*_3*z*_ symmetry locating on three planes (signed as *m*_Γ–*M*–*L*–*A*_) surrounding the Γ point. While SOC is taken into consideration, the band crossings consisting of these node-lines will be gapped, and the P$\overline{3}$c1 YH_3_ becomes a strong topological insulator with Z_2_ indices (1,000). At last, we have calculated the surface states of this system to verify its topological properties. We think our predictions should be helpful to identify the real ground state of YH_3_ in experiments in the future.
